# In-Depth Characterization of Secondary Phases in Cu_2_ZnSnS_4_ Film and Its Application to Solar Cells

**DOI:** 10.3390/nano9060855

**Published:** 2019-06-05

**Authors:** Xianfeng Zhang, Hongde Wu, Engang Fu, Yuehui Wang

**Affiliations:** 1 Zhongshan Institute, University of Electronic Science and Technology of China, Zhongshan 528402, China; wuhongde2019@gmail.com (H.W.); wangzsedu@126.com (Y.W.); 2 State Key Laboratory of Nuclear Physics and Technology, School of Physics, Peking University, Beijing 100871, China; efu@pku.edu.cn; 3 Guangdong Engineering-Technology Research Center of Nano-Photoelectric Functional Films and Devices, Zhongshan 528402, China

**Keywords:** Cu_2_ZnSnS_4_ solar cell, secondary phase, X-ray photoelectron spectroscopy, conversion efficiency

## Abstract

Secondary phases are common in Cu_2_ZnSnS_4_ (CZTS) thin films, which can be fatal to the performance of solar cell devices fabricated from this material. They are difficult to detect by X-Ray diffraction (XRD) because of the weak peak in spectra compared with the CZTS layer. Herein, it was found that in-depth elemental distribution by a secondary ion mass spectroscopy method illustrated uniform film composition in the bulk with slight fluctuation between different grains. X-ray photoelectron spectroscopy (XPS) measurement was conducted after sputtering the layer with different depths. An Auger electron spectrum with Auger parameter were used to check the chemical states of elements and examine the distribution of secondary phases in the CZTS films. Secondary phases of CuS, ZnS and SnS were detected at the surface of the CZTS film within a 50-nm thickness while no secondary phases were discovered in the bulk. The solar cell fabricated with the as-grown CZTS films showed a conversion efficiency of 2.1% (*V*_oc_: 514.3 mV, *J*_sc_: 10.4 mA/cm^2^, *FF*: 39.3%) with an area of 0.2 cm^2^ under a 100 mW/cm^2^ illumination. After a 50-nm sputtering on the CZTS film, the conversion efficiency of the solar cell was improved to 6.2% (*V*_oc_: 634.0 mV, *J*_sc_: 17.3 mA/cm^2^, *FF*: 56.9%).

## 1. Introduction

The kesterite Cu_2_ZnSn(S,Se)_4_ (CZTSSe) semiconductor is recognized as a promising candidate for photovoltaic applications because it uses earth abundant elements only [1,2,3]. A conversion efficiency of 12.6% has been achieved with a nonvacuum method [4]. Cu_2_ZnSnS_4_ (CZTS) has a similar structure and properties to CZTSSe but uses no toxic Se. The highest efficiency of CZTS is about 11% [5]. Currently, the efficiency of CZTS-related solar cells is still far below that of CuInGaSe_2_ (CIGS), which shows the highest efficiency of 23.35% [6]. One important explanation for this is the easy occurrence of secondary phases in the CZTS film due to the narrow existence region of a single kesterite phase according to the phase diagram [5]. Thus, the secondary phases are a particularly serious problem for CZTS layers.

There have been a number of studies that focus on the formation of secondary phases. Some focus on the growth mechanism of CZTS film and give explanations for the formation process of secondary phases [7,8]. Some emphasize how secondary phases affect solar cell performance [9,10]. However, reactions during the fabrication of CZTS films by the nonvacuum method can be significantly different for different approaches due to thermodynamics. Moreover, according to some reports, the secondary phases exist only on the surface of the film [11] and are difficult to detect by X-ray diffraction. It is believed that X-ray photoelectron spectroscopy (XPS) together with Auger electron spectra, because of its high sensitivity, is a good approach to detecting element chemical states.

In this work, CZTS precursors were fabricated by a nonvacuum method followed by a fast-high temperature annealing in a high-sulfur atmosphere. CZTS nanoparticle inks were obtained by a ball milling method and a spin-coating method was used to fabricate CZTS precursors. The specimens were then prepared and characterized immediately after they were taken out of the annealing chamber. To measure the element distribution in the thickness direction, secondary ion mass spectroscopy (SIMS) was conducted. XPS measurement was conducted on the sputtered CZTS films to acquire the in-depth resolved secondary phase distribution. Auger parameter was used to examine the chemical state by measuring the kinetic energies of the element. By checking the element chemical state, the distribution of secondary phases was analyzed. The as-grown film was then applied to a solar cell as the absorber layer and solar cell performance was characterized.

## 2. Experimental Approach

The substrate used in this work was prepared by direct current (DC) sputtering of Mo with a thickness of about 800 nm onto ultrasonically cleaned soda-lime-glass slides. The substrate was then cleaned ultrasonically to remove small particles on the surface before the fabrication of CZTS films by nanoparticle ink. The CZTS ink contains only particles less than 100 nm, which was prepared from CZTS powder by a wet ball-milling method, explained in detail in our previous paper [12]. The concentration of CZTS particles in the ink was adjusted by ethanol to 200 mg/mL. A spin coating process was used to fabricate CZTS precursors. During this process, the substrate (10 × 10 mm) rotated at a speed of 2000 rpm and CZTS ink was dripped on the surface at a speed of 5 μL/min. The obtained CZTS precursor had a thickness of 1–1.5 μm and was annealed in a sulfur atmosphere to improve the grain size and crystallinity in a 15-cm-long ampoule. To conduct CZTS annealing, sulfur powder with a purity of 99.999% was placed together with CZTS film in the ampoule at the two ends. The ampoule was evacuated to around 2.0 × 10^−3^ Pa by a diffusion pump and then sealed. Then, the sample was annealed in an annealing furnace (FP410, Yamato Company, Tokyo, Japan). The furnace temperature was monitored by a thermo sensor and the temperature of the ampoule was monitored by a thermocouple. It was found that the actual annealing temperature deviated less than 1% from the setting value. During the annealing process, the ampoule was heated to 600 °C within 15 min to achieve a high heating rate. After that, the temperature was kept at 600 °C for 20 min and then cooled naturally to around 400 °C over a period of about 15 min to provide the substrate with sulfur atmosphere protection. Then, the ampoule was removed from the annealing furnace and was allowed to cool to less than 200 °C within 5 min in a normal air environment. Then, the as-grown CZTS film was removed from the ampoule and used to conduct characterization immediately. To check the photovoltaic properties of CZTS films, the full solar cell structure was completed as follows: a 50-nm-thick CdS buffer layer was first deposited on the CZTS film by chemical bath deposition. Then, layers of metal-organic chemical vapor deposited i-ZnO (80 nm) and B-doped ZnO (600 nm). Finally, a front-contact Al grid was deposited on top via an evaporation method.

Crystallization of the CZTS film was characterized by X-ray diffraction (XRD, Rigaku, Tokyo, Japan) measurement with a 40-kV voltage and 20-mA current. The composition depth profile of the CZTS film was measured by a secondary ion mass spectrometer (TOF-SIMS, Hitachi, Tokyo, Japan), using a 3-keV primary Cs^+^ ion beam with a sputtering rate of 120 nm/min. To check the depth chemical states of the CZTS films, XPS measurement was conducted by an XPS spectrometer (JPS-9030, JEOL, Tokyo, Japan). An Ar^+^ beam generator attached to the XPS chamber was used to etch the film and the sputtering rate was adjusted to about 5 nm/min with sputtering power of 5 W. The etching rate was determined by sputtering a whole CZTS layer with a known thickness. Before measurement, the surface of the CZTS film was pre-cleaned by sputtering to clear oxidation on the surface. After the pre-clearance, the sample was defined as being in the initiate state of CZTS; that is, etching time 0 min. The sputtering time was selected as 10 min, 1 h, 3 h and 4 h, corresponding to etching depths of 50, 300, 900 and 1200 nm, respectively. After sputtering, X-ray photoelectron spectroscopy (XPS) measurement using a monochromatic Al Kα radiation source (1486.7 eV) was conducted and XPS spectra together with Auger electron spectra (AES) of the element were recorded by JPS-9030 (JEOL, Tokyo, Japan) to check the chemical state. The Mo signal in the spectrum was used to determine interface between the CZTS and Mo layers. Solar cell performance was measured with a 913 CV type current-voltage (J-V) tester (AM1.5) provided by an EKO (LP-50B, EKO, Tokyo, Japan) solar simulator. The simulator was calibrated by a standard GaAs solar cell to determine the standard illumination density (100 mW/cm^2^). The quantum efficiency (QE) of the CZTS solar cell was characterized by a QE-2000 tester (Otsuka Electronics Co., Ltd., Osaka, Japan).

## 3. Results and Discussion

### 3.1. Characterization of the Crystallization of a CZTS Film

**Figure 1:**
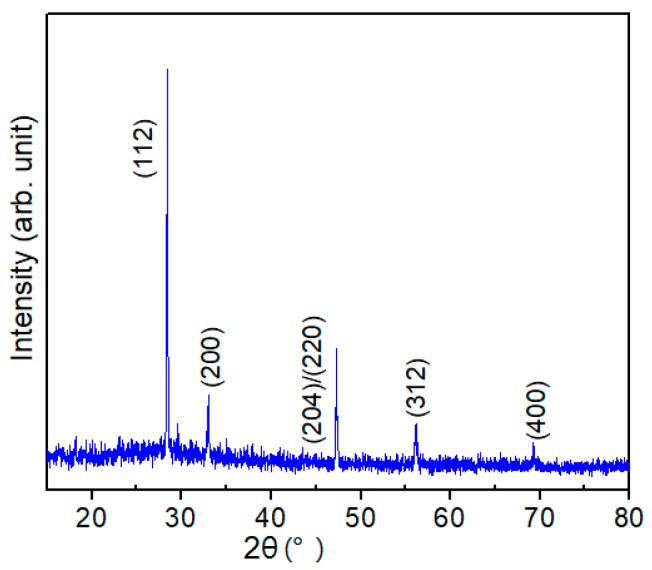
X-ray diffraction (XRD) pattern of a typical Cu_2_ZnSnS_4_ (CZTS) film.

To eliminate the influence of Mo on the spectra, the CZTS film was fabricated on the soda-lime glass (SLG) substrate using the method mentioned in the experimental approach (above) to carry out the XRD measurement. Figure 1 shows a representative XRD pattern of the as-grown CZTS film. In the precursor, only the main peak of kesterite CZTS can be detected [13,14] and no other expected phases, such as ternary Cu-Sn-S phases or binary Cu-S, Zn-S or Sn-S phases, are observed. The film showed a strong (112)-oriented peak, which is common in kesterite CZTS films. The result can be explained in two ways: (1) the CZTS film bulk only contains a CZTS phase and no other phases exist, thus, only peaks of the pure CZTS phase can be observed; (2) other impurity phases also exist in small amounts but are undetectable by XRD measurement or covered by the background noise. To clarify the distribution of the secondary phases, further measurement must be conducted.

### 3.2. In-Depth Elemental Distribution in a CZTS Film

**Figure 2:**
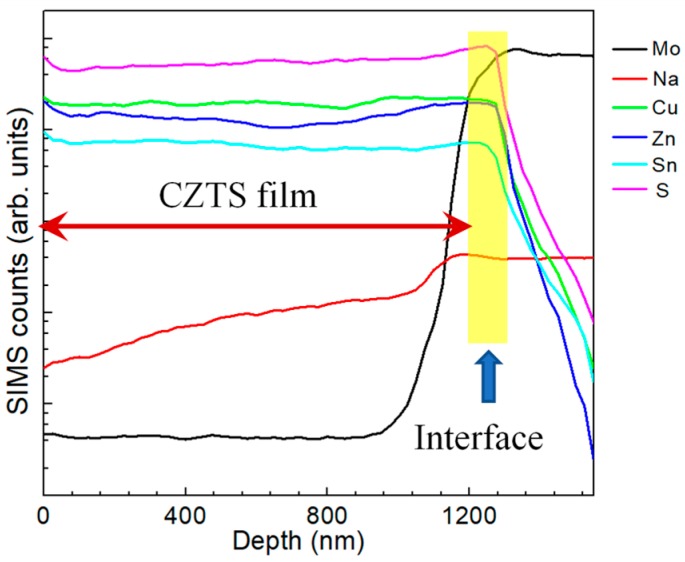
Depth profiles of elements in a CZTS film by secondary ion mass spectroscopy (SIMS) characterization for Mo, Na, Cu, Zn, Sn and S.

Figure 2 illustrates in-depth elemental distribution profiles of a CZTS film by a SIMS method. The yellow area in the figure indicates the interface of the CZTS and Mo layer based on the following: (1) the concentration of Cu, Zn, Sn and S decreased abruptly; (2) the concentration of the Mo element increased significantly. Judging from the figure, the Cu, Zn, Sn and S elements distribute uniformly with small fluctuations in the CZTS bulk. No significant segregation of any element is detected. The fluctuation of elemental distribution is referred to as composition variation between grains. However, all elements show a relatively high concentration near both the front and back surfaces of the CZTS film, indicating relatively high concentrations. The distribution of elements near the front surface is explained by the distribution of secondary phases according to our experimental result in the following section. However, the reason for the high elemental distribution near the back surface remains unknown in our case but a possible explanation is element precipitation. The segregation of S near the CZTS/Mo interface is due to the formation of MoS_2_, which has been widely reported. The concentration of Na gradually increases from the front surface to the back surface of the CZTS film, illustrating the diffusion profile of Na during the fabrication process.

### 3.3. Identification of a Secondary Phase by XPS

**Figure 3:**
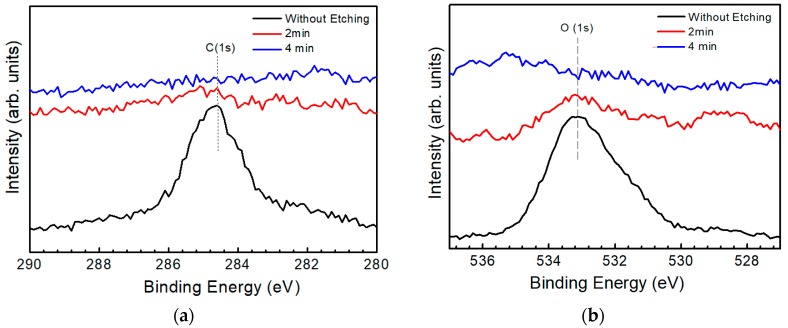
X-ray photoelectron spectroscopy (XPS) spectra of (**a**) carbon and (**b**) oxygen measured at different depths from a CZTS thin film: surface (without etching), 10 nm (2 min), 20 nm (4 min). The spectrum of C was used as the reference and all the peaks were rectified according to the peak of C at a binding energy of 284.6 eV.

Figure 3a,b show the XPS spectra for C and O, respectively, using a binding energy of 284.6 eV of C as the reference to rectify the spectra. The peak at a binding energy of around 284.6 eV is a typical peak of chemisorbed carbon, while a peak at around 532 eV is a typical peak of chemisorbed oxygen [15]. Judging from the figure, C and O atoms mainly exist on the surface of the as-grown CZTS film because of the adsorption effect. As the film was sputtered to a thickness of 10 nm (sputtering time 2 min), the XPS spectrum intensity for both O and C decreased dramatically and no obvious peak was detected, indicating the disappearance of C and O atoms. Adsorption of O and C atoms on the surface was an explanation for the result. 

**Figure 4:**
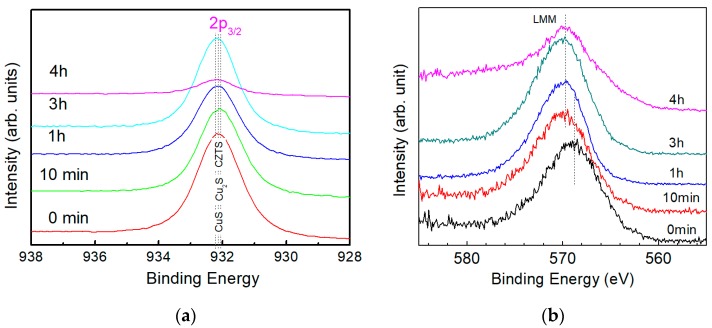
(**a**) XPS spectra of Cu 2p*_3/2_* and (**b**) Auger electron spectra of the Cu LMM of a CZTS film. Dotted lines in (**a**) indicate the peak positions of Cu_2_S, CuS and CZTS and in (**b**) indicate peak positions of the spectra.

For the following sections, samples after surface cleaning to remove oxygen, as explained in the previous section, are defined as initial state of the sample; that is, ‘etching time 0 min.’ Figure 4a shows the XPS spectra for Cu 2p_3/2_ in the CZTS film with etching times of 0 min (without etching), 10 min (50 nm), 1 h (300 nm), 3 h (900 nm) and 4 h (1.2 μm). A single peak around 932.2 eV was observed for all samples and with an increase in etching time, the peak shifted slightly toward a higher binding energy. When the etching time is less than 3 hours, XPS spectra show strong peaks indicating a large amount of Cu compounds. When the sample is etched for 4 hours, the peaking intensity decreases dramatically, which is used to determine the back surface of the CZTS film. During the annealing process for CZTS, secondary phases form easily because of the decomposition of the CZTS films [16]. It is reported that the binding energies of Cu 2p_3/2_ for possible Cu compounds in CZTS film are 932.6 eV for Cu_2_S, 932.5 eV for CuS and 932.2 eV for CZTS [17] and the binding energies of different chemical states are illustrated in Figure 4a. However, it is difficult to determine the chemical state in the film by XPS spectra (as shown in Figure 4a) because of small differences in the binding energies for different compounds. Therefore, Auger spectra of Cu LMM is also acquired with the same CZTS film used to conduct XPS spectra, as shown in Figure 4b.

An Auger parameter cancels the influences of electrical charging at the surface and combines the effects of XPS and AES analysis. Thus, it is effective for separating peaks with similar binding energies in the XPS spectra. Calculation of an Auger parameter is shown in Equation (1) [18].

Auger Parameter α = Binding Energy + Kinetic Energy,(1)

The binding energy (BE) is obtained from the XPS spectra of an element while the kinetic energy (KE) is calculated from the Auger spectra of the element. It is important to state that the BE and KE are both referred to as the Fermi level.

In the case of Cu,

α_Cu_ = BE_(Cu 2p3/2)_ + [hυ−E_(A-Cu LMM)_], (2)

where BE_(Cu 2p3/2)_ is the peak energy of Cu 2p_3/2_ in the XPS spectra, hυ is the X-ray energy of Al Kα (1486.6 eV) and E_(A-Cu LMM)_ is the peak position of the Auger spectra of Cu LMM.

**Figure 5:**
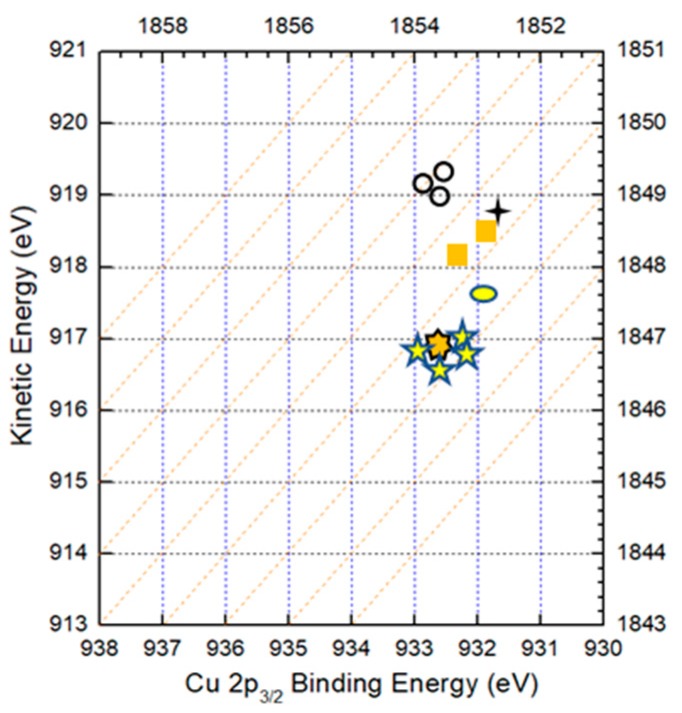
Auger parameters of Cu in Cu metal, CZTS and CuS compounds. The (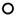), (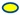), (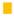) and (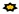) symbols show the reference data of Cu metal, Cu_2_S, CuS and CZTS in the CZTS film, respectively. The (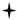) symbol shows the experimental data on the surface in this work. (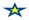) shows the experimental data of the CZTS in the bulk.

Figure 5 shows the Auger parameters of Cu in Cu metal, CZTS and CuS compounds. In the figure, dashed lines represent Auger parameters with an interval of 1 eV. The (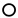), (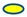), (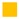) and (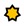) symbols represent reference data of the Cu metal, Cu_2_S, CuS and CZTS film [19,20,21], respectively. The (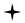) and (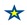) symbols are experimental data of the surface and the bulk, respectively. The Auger parameter of Cu on the surface is quite different from that in the bulk. The parameter of Cu on the surface is near CuS in the CZTS film, while in the bulk it is near CZTS. The result indicates that the surface of the CZTS film is covered by a secondary phase of CuS and no such phase is observed in the CZTS bulk.

**Figure 6:**
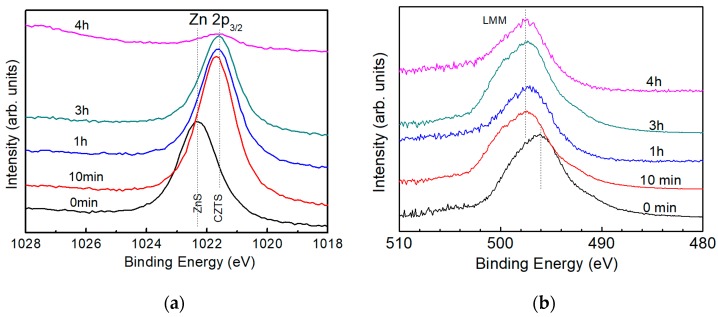
(**a**) XPS spectra of Zn 2p_3/2_ and (**b**) Auger electron spectra of Zn LMM of a CZTS film. Dotted lines in (**a**) indicate the peak positions of ZnS and CZTS and in (**b**) indicate the peak positions of the spectra.

It is reported that the Zn element in the ZnS compound shows a binding energy of 1022 eV [22] and that in pure CZTS, it is around 1022 eV as well [23]. Figure 6a shows the XPS spectra for Zn 2p_3/2_ in a CZTS film with different etching time. Dashed lines in the figure illustrate peak position of the spectra. XPS spectra shows a peak around 1022.8 eV for the surface (etching time: 0 min) and peaks around 1021.4 eV in the bulk, corresponding to ZnS and CZTS, respectively. Thus, it is inferred that the surface of CZTS film is covered by secondary phase of ZnS and no ZnS phase is detected in the CZTS bulk. To further confirm the result, Auger spectra of Zn LMM is acquired with the same CZTS film used to conduct XPS spectra, as shown in Figure 6b.

In the case of Zn, the Auger parameter is calculated as [24]:

α_Zn_ = BE_(Zn 2p3/2*)*_ + [hυ−E_(A-Zn LMM)_], (3)

where the E_(A-Zn LMM)_ is the binding energy of the Auger electron spectra, as shown in Figure 6a.

**Figure 7:**
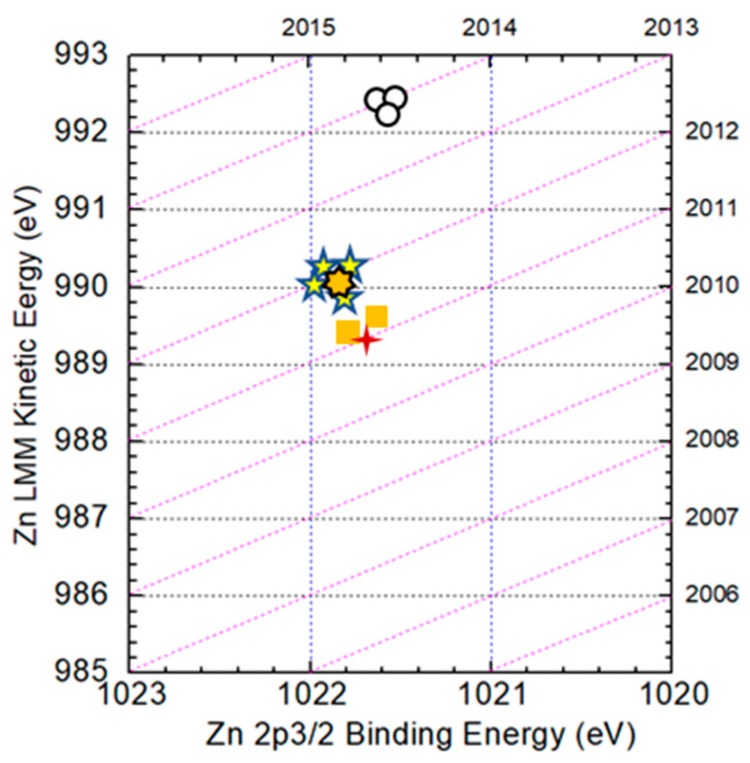
Auger parameters of Zn in Zn metal, CZTS and ZnS compounds. The (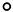), (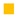) and (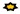) symbols show reference data of Zn metal, ZnS and CZTS in CZTS film, respectively. The (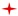) symbol shows experimental data on the surface in this work. (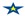) shows experimental data in the bulk of a CZTS film.

Figure 7 shows the Auger parameters of Zn in Zn metal, CZTS and ZnS compounds. In the figure, dashed lines represent Auger parameters with an interval of 1 eV. The (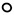), (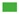) and (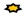) symbols correspond to reference data of Zn metal, ZnS and CZTS film [25,26,27], respectively. The (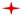) and (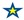) symbols are experimental data of the surface and the bulk, respectively. Judging from the figure, the Auger parameter of Zn on the surface is nearly that of ZnS, while in the bulk it is nearly that of CZTS. The result indicates that the secondary phase of ZnS mainly exists on the surface and in the bulk, ZnS is not detected, which is consistent with a previous result.

**Figure 8:**
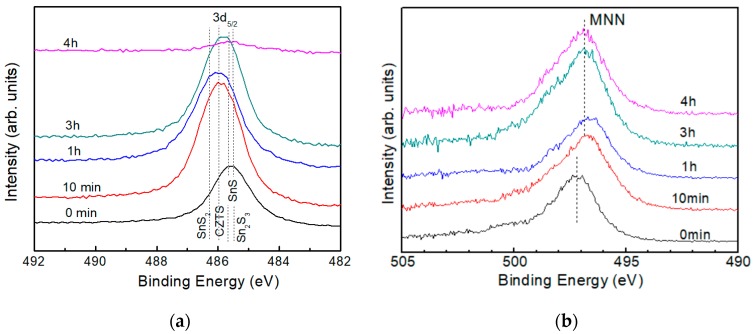
(**a**) XPS spectra of Sn 3d_3/2_ and (**b**) Auger electron spectra of Sn MNN of a CZTS film. The dotted lines in (**a**) indicate peak positions of Sn in SnS compounds and CZTS and in (**b**) indicate peak positions of the Auger electron spectra.

Figure 8a shows the XPS spectra for Sn3d_3/2_ in a CZTS film with different etching times. Dashed lines indicate the peak positions of possible SnS compounds in CZTS films. Binding energies of 485.8, 485.5, 486.5 and 486.3 eV correspond to Sn_2_S_3_, SnS, SnS_2_ and CZTS [28,29], respectively. Although identification of the chemical state in the film is difficult because of the small differences between the binding energies of different peaks, it is clear that chemical states at the surface and in the bulk of CZTS are different. The XPS spectra show a main peak around 485.8 eV at the surface and peaks around 486.0 eV under the surface. Therefore, it is inferred that the surface of the CZTS bulk is covered by a thin layer of an SnS compound. To further confirm our conclusion, Auger electron spectra was acquired, as shown in Figure 8b.

**Figure 9:**
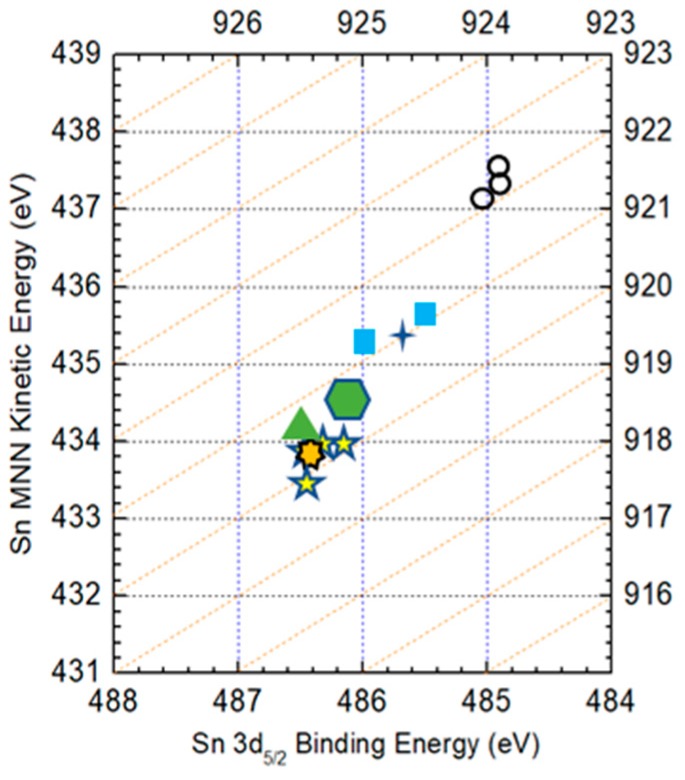
Auger parameters of Sn in Sn metal, CZTS and SnS compounds. The (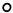), (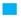), (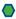) (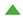) and (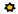) symbols show the reference data of Sn metal, SnS, Sn_2_S_3_, SnS_2_ and CZTS in a CZTS film, respectively. The (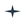) symbol shows experimental data on the surface in this work. (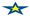) shows experimental data in the bulk of a CZTS film.

In the case of Sn, the Auger parameter is calculated as [30]:

α_Sn_ = BE_(Sn 3d5/2*)*_ + [hυ−*E*_(A-Sn MNN)_], (4)

where, E_(A-Sn MNN)_ is the binding energy of Sn 3d_5/2_ obtained from the Auger electron spectra. The reference Auger parameter of Sn in different chemical states and the experimental data in our CZTS film are drawn together in Figure 9. Dashed lines in the figure represent Auger parameters with an interval of 1 eV. The (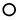), (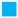), (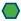), (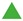) and (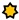) symbols represent the reference data of Sn metal, SnS, Sn_2_S_3_ and CZTS in a CIGS film [25,29,31], respectively. The (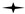) and (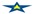) symbols are the experimental data of the surface and the bulk, respectively. The Auger parameter of Sn on the surface is nearly that of the SnS compound, while in the bulk it is nearly that of CZTS, Sn_2_S_3_ and SnS_2_. According to the XRD result, the main structure of the film is CZTS, which shows that the bulk is CZTS but not an SnS compound. Thus, it can be concluded that the surface of the CZTS film is covered by a secondary phase of SnS but no such secondary phase is observed in the bulk, which accords with our previous result. Although it has also been reported that ternary compounds such as Cu_2_SnS_3_ and Cu_3_SnS_4_ may also exist on the surface [32], they were not identified in the XPS spectra in this work.

**Figure 10:**
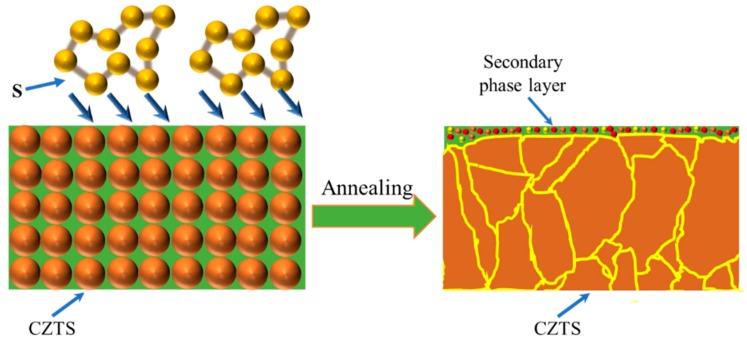
Schematic of the CZTS growth process and formation of secondary phases.

Figure 10 shows a schematic of the growth process of a CZTS film. During annealing, the grain size of the CZTS film gradually grows and secondary phases precipitate on the surface of the film. The total thickness of the secondary phase layer is several tens of nanometers and consists of CuS, ZnS and SnS compounds.

**Figure 11:**
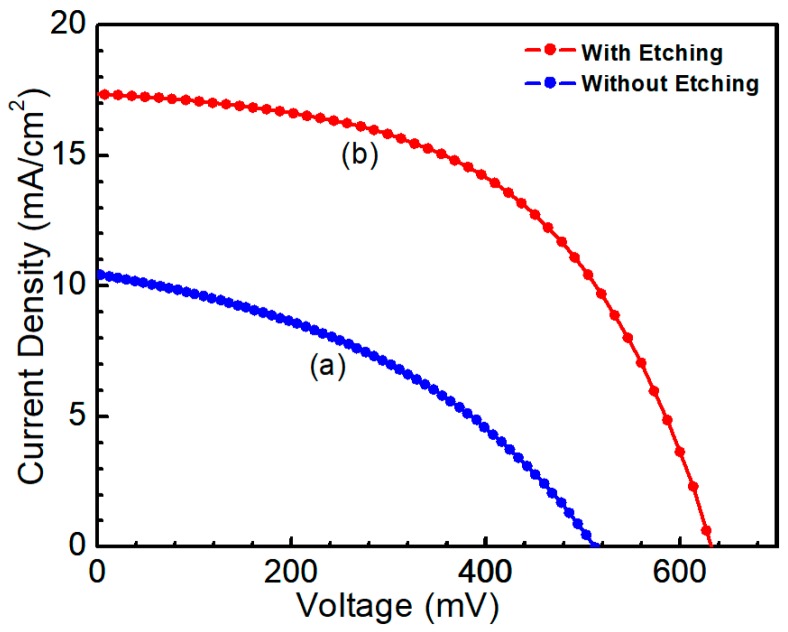
J-V curve of CZTS solar cells (**a**) without etching (**b**) etching for 10 min.

To improve CZTS/CdS interface quality, the CZTS film was etched for 10 min (50 nm), aiming at reducing the influence of the secondary phase on solar cell performance. The CZTS films before and after etching were fabricated with other layers to complete the solar cell structures. Solar cell performance was evaluated under standard conditions with an irradiation of 100 mW/cm^2^. The intensity of the solar simulator was calibrated by a high-precision monocrystalline Si solar cell to achieve a standard illumination. Figure 11 shows the J-V curve of CZTS solar cells and the area was 0.2 cm^2^. The photovoltaic device without etching exhibited a conversion efficiency of 2.1%, with open circuit voltage (*V*_oc_) = 514.3 mV, short circuit current density (*J*_sc_) = 10.4 mA/cm^2^ and fill factor (*FF)* = 39.3%. For the CZTS absorber with etching, the solar cell showed an efficiency of 6.2%, with *V*_oc_ = 633.3 mV, *J*_sc_ = 17.3 mA/cm^2^ and *FF* = 56.9%.

**Figure 12:**
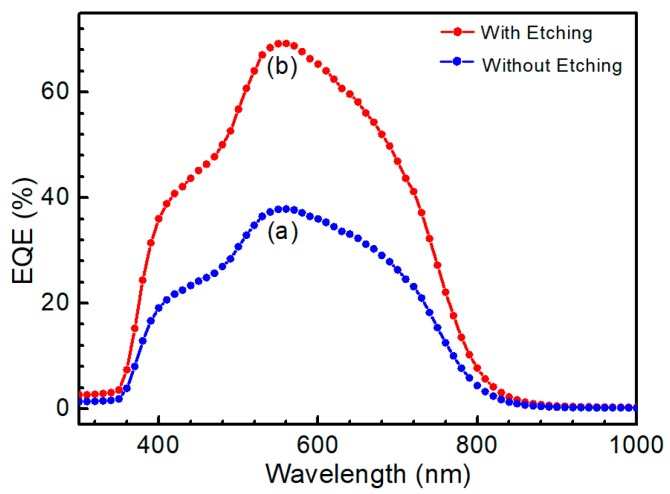
Quantum efficiency of CZTS solar cells (**a**) without etching (**b**) etching for 10 min.

Figure 12 shows the external quantum efficiency (EQE) curve of the CZTS solar cell. After etching, the QE was significantly improved, as shown in the figure. The QE curve exhibits an abrupt drop in the infrared region around 770 nm, which was the CZTS absorption edge. Thus, the calculated bandgap of the CZTS films was about 1.61 eV. The features near 510 and 380 nm corresponded to the absorption edges of the CdS and ZnO layers [33], respectively, which was common when using the respective CdS buffer and ZnO window layers. Based on the EQE data of a solar cell, *J*_sc_ was calculated as [34]:

(5) $$J_{sc}=q\int_{0}^{\infty }QE\left(E\right)b_{s}\left(E,T_{s}\right)dE$$

where, *q* is the elementary charge, *QE* is quantum efficiency and *b_s_* is solar flux or irradiation. For air mass 1.5, the data are available from Ref. [35]. Based on Equation (5), Figure 12 and the solar irradiation spectrum, *J*_sc_, of the CZTS solar cell was calculated as 8.4 mA/cm^2^ for a CZTS solar cell without etching and 15.5 mA/cm^2^ for a solar cell with etching. Because the J-V curve represents the real performance of a photovoltaic device, the slight deviation of *J*_sc_ calculated from the QE curve can be explained as follows: the QE measurement is carried out at a single wavelength with much lower intensity than that of a one sun irradiation.

## 4. Conclusions

A CZTS film was obtained by annealing a CZTS nanoparticle precursor in an S atmosphere. Elemental distribution of the film was uniform with slight fluctuation judging from the SIMS result. Secondary phases that could not be distinguished by XRD based techniques were identified by an XPS measurement. In-depth chemical states of elements were checked by combing Auger electron spectra and an Auger parameter at different depths of the film. When CZTS films were annealed in the S atmosphere, the surface of the films existed a secondary phase layer composed of CuS, ZnS and SnS while the whole film below the surface was in a CZTS phase. To remove the secondary phase, the CZTS film was etched to a depth of 50 nm and fabricated with other layers to complete the solar cell structure. The conversion efficiency of the CZTS solar cell was improved from 2.1% (*V*_oc_: 514.3 mV, *J*_sc_: 10.4 mA/cm^2^, *FF*: 39.3%, area: 0.2 cm^2^) to 6.2% (*V*_oc_: 634.0 mV, *J*_sc_: 17.3 mA/cm^2^, *FF*: 56.9%, area: 0.2 cm^2^) under standard solar illumination.
